# Relational Victimization and Video Game Addiction among Female College Students during COVID-19 Pandemic: The Roles of Social Anxiety and Parasocial Relationship

**DOI:** 10.3390/ijerph192416909

**Published:** 2022-12-16

**Authors:** Gengfeng Niu, Siyu Jin, Fang Xu, Shanyan Lin, Zongkui Zhou, Claudio Longobardi

**Affiliations:** 1 Key Laboratory of Human Development and Mental Health of Hubei Province, School of Psychology, Central China Normal University, Wuhan 430079, China; 2 Key Laboratory of Adolescent Cyberpsychology and Behavior (CCNU), Ministry of Education, Wuhan 430079, China; 3 Center for Research on Internet Literacy and Behavior, Central China Normal University, Wuhan 430079, China; 4 Collaborative Innovation Center of Assessment toward Basic Education Quality, Central China Normal University Branch, Wuhan 430079, China; 5 Department of Psychology, University of Turin, 10124 Torino, Italy

**Keywords:** video game addiction, relational victimization, social anxiety, parasocial relationship

## Abstract

Video game addiction, a common behavioral problem among college students, has been more prominent during the COVID-19 pandemic; at the same time, females’ video game usage has also attracted considerable research attention. Against this background and under the perspective of social interaction, this study aimed to examine the relationship between relational victimization and video game addiction among female college students, as well as its underlying mechanism—the mediating roles of social anxiety and parasocial relationships with virtual characters. Female college students (*N* = 437) were recruited to complete a set of questionnaires voluntarily in June 2022. Through the mediating effect analysis, the results found that (1) relational victimization was positively associated with female college students’ video game addiction; (2) social anxiety and parasocial relationships with virtual characters could independently mediate this relation; (3) social anxiety and parasocial relationships with virtual characters were also the serial mediators in this association. These findings not only expand previous studies by revealing the social motivation of video game usage and the underlying mechanism accounting for video game addiction, but also provide basis and guidance for the prevention and intervention of video game addiction in the current context of the COVID-19 pandemic.

## 1. Introduction

Nowadays, with the prevalence of Internet technology, video games have become a popular mode of entertainment worldwide, with great appeal to young teenagers such as college students [1]. There were nearly 2 billion video game players all around the world in 2015 and this number is expected to rise to over 3.3 billion by 2024 [2]. In China, the number of game players had reached 666 million in 2021. With this trend, the phenomenon of excessive video game use also has been increasingly prominent, which has been defined as “Internet Gaming Disorder” in the American Psychiatric Association’s Diagnostic and Statistical Manual of Mental Disorders (DSM-5, APA) [3] to describe this out-of-control video game behavior. It has been well-established that video game addiction can cause various deleterious effects on individuals, such as depression, fatigue, loneliness, low self-esteem, and impulsion [4]. Against this background, examining the risk factors underlying video game addiction has been a focus of relevant studies and interpersonal factors were found to be an important predictor [5]. At the same time, in spite of the fact that males, who account for the majority of video game users, are always considered to be the main targeted players of game products, female players are increasing and have been gaining an increasingly important role in the game market [6]. In China, females have gradually become active consumers in the domestic video game market and the number of female players has exceeded 300 million, accounting for approximately half of the total number of players [7]. Thus, more importance should be attached to the factors influencing females’ video game use (including video game addiction.

In addition, under the background of the COVID-19 pandemic in recent years, college students’ daily life, especially social relationships, has been greatly interrupted [8]. In addition to the psychological distress caused directly by the COVID-19 pandemic, they have been asked to be obliged to stay on campus. Offline gathering entertainment and physical contact were also restricted, which may further aggravate their psychological distress, and negative interpersonal experiences (e.g., interpersonal conflict) may also become increasing in this context [9]. Along with this reality, college students have to spend more time on the Internet to study, contact others, and for entertainment [10,11]; correspondingly, playing video games has become a main way for students to entertain themselves and deal with stress [12]. Thus, this means college students tend to be more engaged in video games, and even increase the risk of video game addiction. Relevant studies also found that the risks of addiction have been growing during the pandemic: about half of people reported increased dependence on Internet use after the COVID-19 pandemic [11], and over one-third of game players reported spending increased time on gaming per day during the lockdowns [13]. Based on these evidences, this study aimed to examine the association between relational victimization and video game addiction among female college students and its underlying mechanism, under the background of the COVID-19 pandemic.

### 1.1. Relational Victimization and Video Game Addiction

It has been well established that stressful life events or adversities were closely associated with video game addiction [14,15]. According to cognitive-behavioral theory, experiencing negative life events, in particular, would make people at a higher risk of being an addict [16,17], and various sources of stress (e.g., students’ physical problems, mental maladjustment, and academic difficulties) may contribute to video game addiction [18,19]. In particular, the negative environment and interpersonal stress (e.g., parental psychological control, and peer victimization) could increase the risk of game addiction [20,21]. Peer victimization, which has been defined as being repeatedly harmed or exposed to aggressive behavior from peers, is a common adversity among students and is a great negative social experience for individuals that results in great harm to their health and well-being [22]. A review demonstrated that peer victims typically lead to various adverse outcomes, such as anxiety and depression, and experience poor self-esteem, loneliness, and isolation [23]; at the same time, there exist gender differences in peer victimization, and female students are more likely to experience relational victimization [23], which is also more hurtful for female students [24,25]. Previous studies indicated that female students usually experienced more negative relational changes during the COVID-19 pandemic [26]. Although relational victimization is an important predisposing factor for video game addiction, the internal mechanism of the relationship is unclear yet. Thus, this study aimed to examine the association between relational victimization and video game addiction among female college students and its underlying mechanism, and it was hypothesized that:

**Hypothesis** **1.** *Relational victimization would be positively related to video game addiction among female college students.*

### 1.2. The Role of Social Anxiety

Social anxiety is defined as an excessive and persistent fear in social or performance situations that are either avoided or painfully and reluctantly endured [3]. It’s an indicator of one’s quality of social relationships, and people who feel socially anxious have more difficulties establishing and maintaining positive relationships and experience more negative social experiences in real life, which may induce other adaptation problems, including behavioral problems (e.g., addictive behaviors) [27]. According to the psychological-decompensation model, socially anxious individuals may look for secure social opportunities or settings, such as online social media or YouTube, to compensate for the lack of relationships in real life, and thus become addicted to these opportunities in the end [28,29]. Barr and Copeland-Stewart [30] also found that the video game was a convenient alternative outlet for adolescents with social anxiety during the pandemic due to the fact that it provided cognitive stimulation and opportunities to socialize online. Nowadays, social elements are regarded as critical to video game enjoyment [31], and playing video games is considered a social accommodator for the insecurely attached [32]. Therefore, it was hypothesized that social anxiety is positively correlated with video game addiction.

Negative social interactions are crucial predisposing factors of social anxiety [33]. Previous studies have shown that peer victimization, as a common negative social experience among adolescents [34,35], was a predictor of social anxiety over time, with the most robust results found for relational victimization [36,37]. Moreover, individuals’ perceived severity is highly associated with the feelings of anxiety [38], and recent studies have shown that COVID-19’s severity could also predict individuals’ social anxiety [39]. As a serious consequence of relational victimization, social anxiety is also a predictor of video game addiction [40,41]. According to cognitive-behavioral theory, social anxiety is an underlying psychopathology in overuse of the internet [16]. Thus, it was hypothesized that:

**Hypothesis** **2.** *Relational victimization would predict female college students’ video game addiction via the mediating role of social anxiety.*

### 1.3. The Role of Parasocial Relationships

In addition, the experience in video games (such as escaping from reality or socializing with others) is another factor influencing video game addiction [42,43]. A parasocial relationship is a one-sided connection to virtual characters [44], which is a common experience in video games. As video games always provide various virtual characters with which to interact, it seems to be a favorable environment for the development of parasocial relationships. Although a parasocial relationship is an attractive experience in video games, it can be problematic in some cases [45], especially in cases when the parasocial relationships with virtual characters in video games may be a compensation for the social relations in real life [46,47]. Based on the parasocial compensation hypothesis, the need to belong could be satisfied with parasocial relationships by compensating for the lack of relationships in real life [48,49,50]. However, according to the psychological-decompensation model, extreme parasocial relationships form pathological compensation are not adequate for students and can lead to the developmental deviation [51]. Previous studies have shown that establishing parasocial relationships with favorite characters in internet fiction leads to addictive behavior [47], and many empirical studies have also indicated that parasocial relationships could lead to addictive behavior by increasing flow experience and urge [29,52]. Thus, experiences of parasocial relationships in video games may encourage individuals to become more involved in it. 

The parasocial compensation hypothesis also indicated that parasocial relationships are associated with unsatisfying social life and social deficits [48]. Previous studies found that parasocial relationships support social function [53] and can be resources to substitute or compensate for close relationships and social belonging [46,54]. In addition, existing studies also indicated that peer victimization is a predictor of mobile social addiction [55,56], which means that people whose social life is hindered may find some controllable ways to compensate. As discussed above, relational victimization is a common adversity for female college students who will face the situation of lacking social contacts [23]. According to the parasocial compensation hypothesis, parasocial relationships can provide resources to compensate for the lacking needs for these students and, thus, can boost individuals’ engagement in video game usage and increase the risk of video game addiction. Thus, it was hypothesized that:

**Hypothesis** **3.** *Relational victimization would predict female college students’ video game addiction via the mediating role of parasocial relationships.*

### 1.4. The Serial Mediating Effect of Social Anxiety and Parasocial Relationships

**Figure 1:**
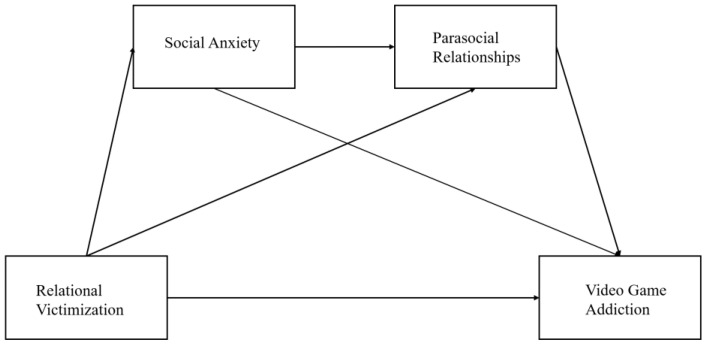
The hypothetical figure of social anxiety and parasocial relationships between relational victimization and video game addiction.

Moreover, social anxiety is also closely associated with parasocial relationships. Social anxious individuals often experience negative relationship quality and experience, and these hinder the realization of interpersonal communication and belongingness needs [57]. Recently, some empirical studies have shown that social anxiety and interpersonal alienation may lead to strong parasocial relationships [29,47]. Parasocial relationships constitute an extension of one’s social circle for most people; however, individuals with high level of social anxiety would seek a safe social environment in order to satisfy their social needs [58]. They are considered to develop more intense parasocial relationships with virtual characters because it can provide safe environments for interpersonal experiences based on the parasocial compensation hypothesis [48]. Existing research has shown that face-to-face social engagement with real friends was considerably reduced during COVID-19 social distancing, but parasocial closeness with virtual characters increased over time [59], which may provide indirect evidence for their relation. Based on the related theoretical and empirical research, the following hypothesis was proposed:

**Hypothesis** **4.** *Social anxiety and parasocial relationship operated as serial mediators between relational victimization and female college students’ video game addiction (Figure 1).*

Combining the parasocial compensation hypothesis with psychological-decompensation model, the present study constructed a serial mediation model to examine the mechanisms underlying the relationship between relational victimization and video game addiction among female college students.

## 2. Materials and Methods

### 2.1. Participants

Through convenience sampling, 464 female students from an educational college in central China were recruited to voluntarily participate in this study in June 2022, when college students were required to live in their dormitory on campus unless necessary after March because of the COVID-19. In the end, 437 female college students completed the survey, whose ages ranged between 16 and 25 years (M = 19.41; SD = 1.38). The participants frequently play video games with a score of 4.5 on a 6-likert scale (1 = Never, 6 = Everyday; M = 4.5; SD = 1.24); more than half of them like to play King of Glory, and the others like to play mobile romantic video games, Genshin impact, and so on, all of which provide opportunities to interact with virtual characters. The study was approved by the ethics committee of the author’s organization, and the participants were informed of the principles of anonymity, independence, and confidentiality.

### 2.2. Measurement

#### 2.2.1. Relational Victimization

The relational victimization was measured by the Chinese version of the Olweus Bully/Victim questionnaire (OBVQ) [60,61]. The relational victimization subscale (5 items) was adopted to measure the degree to which female college students were actively isolated or manipulated socially (e.g., “how often do others tell you they will stop liking you unless you do what they say?”). Items are rated on a 5-point Likert scale anchored by 1 (never) to 5 (more than 5 times), with higher score indicating a higher level of relational victimization. This study reported adequate internal reliability with Cronbach’s α = 0.92.

#### 2.2.2. Social Anxiety

Social anxiety was assessed by the Chinese version of Social Anxiety Scale [62,63]. This scale has 6 items (e.g., “it takes me time to overcome my shyness in new situations.”), and participants were asked to respond on a 5-point Likert scale, ranging from 1 (strongly disagree) to 5 (strongly agree). The average score of these items was computed as the final score, with a higher score indicating a higher level of social anxiety. The present sample revealed acceptable internal reliability with Cronbach’s α = 0.79.

#### 2.2.3. Parasocial Relationships with Virtual Characters

Parasocial relationship was measured using the Chinese 11-item version of the Parasocial Relationship Scale [64,65,66], which contains three dimensions: parasocial cognition, parasocial affection, and parasocial behavior (e.g., “I feel happy when the characters are happy.”). The original scale was designed to measure the parasocial relationship of readers with manga characters, and the wording has been revised to adapt to the video game context. For example, “manga characters” was replaced by “video game characters.” Items are rated on a 5-point Likert scale (1 = strongly difficult, 5 = easier), and an average score of these items was computed with higher scores indicating a higher level of parasocial relationship towards virtual characters in video games. The Cronbach’s α of this scale was 0.93.

#### 2.2.4. Video Game Addiction

Video game addiction was assessed with a widely used 7-item Game Addiction Scale (GAS) [67,68]. Several studies find that the Chinese version of this scale is a suitable assessment tool with adequate reliability and validity to assess a video gaming disorder among Chinese college students [67]. Items (e.g., “have you ever wanted to play video games all day?”) were rated on a 5-point Likert scale (1 = never, 5 = usually). An average score of these items was computed, with a higher score indicating a higher level of video game addiction. The Cronbach’s α in the present study was 0.92.

### 2.3. Statistical Analyses

IBM SPSS 26.0 and the PROCESS macro [69] were adopted to analyze the data. First, confirmatory factor analysis for a single factor was conducted to examine the extent of common method bias because all the data were collected via questionnaires. The result showed that the variation of the first factor was 22.3%, less than 40% of the critical standard [70,71], indicating no serious common method bias in the present study. All continuous variables were standardized. Then, the descriptive statistics, mean differences, and Pearson’s correlation analysis for the main study variables were computed. Afterwards, the present model was tested by Hayes PROCESS macro for SPSS (Model 6), and all mediation analyses were conducted using it with 5000 bootstraps sample. The bootstrap method generated 95% bias-corrected confidence intervals (CI) for all the indexes and indirect effects. If zero is not included in the 95% CI, the effects were regarded as significant.

## 3. Results

### 3.1. Descriptive and Correlation Analysis

The results of descriptive statistics and Pearson’s correlation analysis for all the observed variables that relational victimization, social anxiety, parasocial relationship with virtual characters, and video game addiction were shown in Table 1. Previous studies have revealed that age might affect the results [72]. For this reason, age was as the covariate variable for a more rigorous model. Relational victimization was positively correlated with social anxiety (r = 0.20, *p* < 0.001), parasocial relationship with virtual characters (r = 0.28, *p* < 0.001), and video game addiction (r = 0.30, *p* < 0.001). Social anxiety was positively correlated with parasocial relationship (r = 0.29, *p* < 0.001) and video game addiction (r = 0.35, *p* < 0.001). Parasocial relationship was positively correlated with video game addiction (r = 0.44, *p* < 0.001).

### 3.2. Testing for the Proposed Model

It was hypothesized that social anxiety and parasocial relationships with virtual characters in video games would mediate the relationship between relational victimization and video game addiction. The PROCESS macro for SPSS (model 6) by Hayes was used to examine the possible mediating roles of social anxiety and parasocial relationships with virtual characters. The main results of the serial mediation analysis are presented in Table 2 and Figure 1. After controlling for the age, relational victimization was positively associated with video game addiction among female college students (β = 0.17, *p* < 0.001). Thus, the Hypothesis 1 was supported. It also can be seen that relational victimization was positively associated with social anxiety (β = 0.19, *p* < 0.001), and social anxiety was positively associated with video game addiction (β = 0.21, *p* < 0.001). Social anxiety served a mediating role between relational victimization and video game addiction and, therefore, Hypothesis 2 was supported. In addition, relational victimization was positively associated with parasocial relationships (β = 0.23, *p* < 0.001), and parasocial relationships were positively associated with video game addiction (β =0.33, *p* < 0.001) after controlling for age. Therefore, the Hypothesis 3 was supported.

Then, a bootstrap program was adopted to further test and compute the mediating effects. As shown in Table 3, all three paths did not include zero in the 95% confidence interval, which means that the mediating path was significant, and Hypothesis 4 was supported. The total mediating effect value was 0.13, and three mediating paths accounted for 44.50% of the total effect of relational victimization on video game addiction, which is the ratio of indirect effects to the total effects.

## 4. Discussion

In the current information era, with the increasing popularity of video games, the problem of video game addiction has also been increasingly prominently, especially in the context of the COVID-19 pandemic. The pandemic has taken a toll on interpersonal relationships, and females’ use of video games is increasing and needs more attention. Against this background, this study aimed to examine the association between relational victimization and female college students’ video game addiction, as well as its internal mechanisms. The results indicated that the experience of relational victimization was positively associated with female college students’ video game addiction through the mediating effects of social anxiety and parasocial relationships with virtual characters. The mediating effects include three mediating paths: the separate mediating effects of social anxiety and parasocial relationships, as well as the serial mediating effect of them. Specifically, parasocial relationships may play a relatively more important role in the internal mediating mechanism. This study may expand previous studies and have significant theoretical and practical significance.

### 4.1. The Effect of Relational Victimization on Video Game Addiction

Interpersonal relationship is an important factor affecting individual developments and adaptations [73]. Positive interpersonal interactions and relationships, in particular, can promote developments and adaptations, while negative interpersonal interactions and relationships will lead to adverse consequences, such as anxiety, depression, and so on [74]. Firstly, the results found that relational victimization was significant and positively associated with video game addiction among female college students, which is consistent with previous studies conducted in the general population or in males [24,75]. Regarding Internet use, it has been well-established that negative life events are distal causes of pathological Internet use based on the Cognitive-Behavioral model [16]. Relational victimization is a common stressful social experience for college students [76], especially for females. It would induce serious psychological distress and further lead to adverse adaptation outcomes, such as mobile social addiction, video game addiction, and other problematic behaviors [24,55]. For female college students, the negative effects of relational victimization are even more pronounced [77]. Moreover, relational victimization is hidden in most of the cases. The emotional and psychological damage and abuse may be more traumatic to female college students’ development and lead to more stress and negative emotional experience [78]. In the context of the current COVID-19 pandemic, the importance of interpersonal relationships has become more prominent, but the pandemic has hindered face-to-face interpersonal interaction and brought more negative experiences [59]. One point that needs special attention is that due to the school lockdowns, it may be difficult for the “recipients” of relational bullying to escape from the “perpetrators” on campus, which may aggravate the negative effects of relational victimization. Previous studies have indicated that individuals tend to adopt negative coping measures (e.g., problematic mobile phone use, problematic SNS use, and game addiction) when faced with bullying [17,24,79]. When encountered with relational bullying, female college students may turn to video games with the aim of escaping from bullying in the real world or dealing with negative feelings [58]. Thus, relational victimization was positively associated with female college students’ video game addiction.

### 4.2. The Mediating Roles of Social Anxiety and Parasocial Relationships

The further mediating analyses found that social anxiety and parasocial relationships could mediate the relationship between relational victimization and video game addiction. Regarding the simple mediating effects of social anxiety and parasocial relationships, they were consistent with the main points of the Cognitive-Behavioral model and the parasocial compensation hypothesis [16,48]. There are many functions in video games which could meet players’ needs, such as entertainment and interpersonal interaction [80]. Especially with the development of video games technology, the function of interpersonal interaction has become more and more prominent and is now one of the main motivations for people to play video games [81]. At the same time, it is also a convenient substitute for groups facing undesirable social experiences in real life [32]. The maladaptive cognition regarding interpersonal relationships in real life and in virtual world would be reinforced, and individuals are more likely to be addicted to video games [16]. As a negative and stressful experience, relational victimization can be seen as a distal cause and social anxiety can be seen as a proximal cause of video game addiction [16]. Meanwhile, relational bullying may greatly damage individuals’ sense of belongings, and make them hold negative attitudes towards social interaction and others, namely they may become socially anxious because of the relational bullying victimizations [82]. Social anxiety is a predictor for video game addiction. Many previous studies have found that social anxiety is a risk factor for video game addiction due to the limited social interaction required in video games and to the characteristics of the interaction, which are perceived as safe by social anxious players [83,84]. Thus, the individuals frequently encountered with relational victimization may avoid and escape from social interaction in real life, seek compensation in video games, and further become addicted to video games.

This is true for parasocial relationships; the negative feelings and the damaged social relation may push individuals to seek compensation through other means. For example, they may use video games to alleviate the stress and form parasocial relationships with characters in games to compensate for basic needs, especially during the COVID-19 pandemic [48,85]. Parasocial relationships may increase the risk of video game addiction. The human brain is believed to have difficulties in distinguishing real interpersonal relationships from parasocial relationships [86], and some students have built extreme forms of parasocial relationships that have foregone real-life intimate relationships, substituting them for parasocial relationships [87]. Thus, parasocial relationships may lead to excessive video game usage and further video game addiction [80,88]. For example, dependence on parasocial relationships has also been associated with maladaptive behaviors such as social network addiction, internet fiction addiction, or video game addiction [29,47,89].

The current finding is also consistent with the uses and gratification theory. This theory indicated that the level of game use is predicted by the strength of player motivations determining such uses [90] and seeking media for gratifications of companionship is considered to be the strongest motivation predictor for media addiction [91]. In other words, using video games to escape reality, cope with stress, and seek companionship may lead to video game addiction [92]. The present study confirmed that social anxiety and parasocial relationships serve significant mediating roles between relational victimization and video game addiction.

Furthermore, the serial mediating effect of social anxiety and parasocial relationships between relational victimization and video game addiction was also found in the present study, suggesting that parasocial relationships are a more direct factor contributing to video game addiction. This further suggests that game experience and feelings are key factors that influence game use behavior, including addiction [42,93]. Although not all parasocial relationships will definitively lead to negative effects, the extreme forms (e.g., celebrity worshiping) are also associated with maladaptive social behaviors and negative effects on psychological well-being [94,95]. The parasocial compensation hypothesis indicated that individuals who feel lonely, socially isolated, and socially anxious tend to develop higher levels of parasocial relationships [48]. In particular, those experiencing negative life events and becoming anxious about socializing have a more difficult time establishing positive relationships in real life [96]. However, idealizing parasocial relationships gives them a safe and controllable situation to compensate for satisfying the needs of belongings [47]. Besides, the finding are also consistent with the psychological-decompensation model [51]. To be specific, people who experience relational victimization would be anxious about social interaction, and if they tend to turn to virtual characters in video games for compensation, the development may be blocked, and they will be addicted to video games permanently.

### 4.3. Limitations and Implications

The present findings may have some theoretical and practical implications. Theoretically, this study expanded previous studies by firstly focusing on the female video game user other than the male users. Secondly, these findings deepen our understanding of the influencing factors and underlying mechanisms of video game addiction by integrating the social experience of real life and the specific experience of the game. It may also provide a wider perspective on the influences of relational victimization and the risk factors of video game addiction, under the context of the COVID-19 pandemic. Practically, this study may provide guidance for the prevention and intervention of video game addiction among females, since it is a common social phenomenon, especially during lockdown [13]: Firstly, relational victimization and social anxiety should be taken seriously, and relevant intervention procedures such as social skills training could be adopted to avoid relational victimization and social anxiety; Secondly, though parasocial relationships are an appealing experience and could compensate for the real social relation, individuals should avoid too much engagement into parasocial relationships with virtual characters, and should be encouraged to participate in more offline social interactions.

However, some limitations should also be acknowledged. First, the study only used cross-sectional data. To facilitate the inference of causal effects, longitudinal design or experimental design should be adopted in future research. Second, all the participants were Chinese female college students. Studies suggested that collectivist culture in east Asia places a high value on harmonious interpersonal relationships [97]. Future studies may examine the cultural differences with culture-diverse participants. Third, all participants were recruited to participate into the survey voluntarily, which may cover up the severity of relation victimization and video game addiction. Future studies may collect data from multiple modalities/multiple sources to further examine the mechanism. Fourth, future studies may further examine other underlying mechanisms (e.g., the moderators), especially the protective factors. Combined with the characteristics of video games and the internet, online social capital and online game social migrations may moderate the paths. Players using the internet or online games to make friends with other players or even become friends in real life seems to be a good way to expand their social resources during the pandemic [98].

## Figures and Tables

**Table 1:** Descriptive statistics and correlations of the main studied variables.

Variables	M	SD	2	3	4	5
1. Age	19.41	1.38				
2. Relational Victimization	1.47	0.85	1			
3. Social Anxiety	3.21	0.80	0.20 ***	1		
4. Parasocial Relationship	2.65	0.88	0.28 ***	0.29 ***	1	
5. Video Game Addition	1.83	0.78	0.30 ***	0.35 ***	0.44 ***	1

Notes: *N* = 437. M = mean; SD = standard deviation; *** *p* < 0.001.

**Table 2:** Testing the serial mediation effect of relational victimization and video game addiction.

Outcome	Predictors	*R* ^2^	*F*	*β*	*t*	LLCI	ULCL
SA	Age	0.05	10.61 ***	0.05	1.58	−0.01	0.12
	RV			0.19	4.09 ***	0.10	0.29
PRs	Age	0.14	22.91 ***	0.01	0.37	−0.05	0.08
	RV			0.23	4.99 ***	0.14	0.32
	SA			0.24	5.34 ***	0.15	0.33
VGA	Age	0.27	40.01 ***	0.01	0.49	−0.04	0.07
	RV			0.17	3.80 ***	0.08	0.25
	SA			0.21	4.89 ***	0.13	0.30
	PRs			0.33	7.46 ***	0.24	0.42

Notes: *N* = 437. RV, relational victimization; SA, social anxiety; PRs, parasocial relationships; VGA, video game addiction, LLCI, lower level of confidence interval; ULCI, upper level of confidence interval. *** *p* < 0.001.

**Table 3:** Total, direct, and indirect effects of relational victimization on video game addiction through social anxiety and parasocial relationships.

	Path	*β*	SE	LLCI	ULCI	Relative Value
Total effect		0.30	0.05	0.21	0.39	
Direct effect	RV→VGA	0.17	0.04	0.08	0.25	
Indirect effect	RV→SA→VGA	0.04	0.01	0.02	0.07	13.82%
	RV→PR→VGA	0.08	0.02	0.04	0.11	25.43%
	RV→SA→PR→VGA	0.02	0.01	0.01	0.03	5.25%
Total indirect effect		0.13	0.02	0.09	0.18	44.50%

Note: *N* = 437. RV, relational victimization; SA, social anxiety; PRs, parasocial relationships; VGA, video game addiction.

## Data Availability

The data of this study are available from the corresponding author upon reasonable request.
